# Supplementary material for: Neutrophil activation and clonal CAR-T re-expansion underpinning cytokine release syndrome during ciltacabtagene autoleucel therapy in multiple myeloma
Source: Nat Commun. 2024 Jan 8;15:360. doi: 10.1038/s41467-023-44648-3 (PMC10774397; doi:10.1038/s41467-023-44648-3)
Supplement: Supplementary file 1 — Supplementary Information [file 41467_2023_44648_MOESM1_ESM.pdf]

Supplementary Information

Neutrophil activation and clonal CAR-T re-expansion underpinning cytokine release syndrome during ciltacabtagene autoleucel therapy in multiple myeloma

Shuangshuang Yang, Jie Xu, Yuting Dai, Shiwei Jin, Yan Sun, Jianfeng Li, Chenglin Liu, Xiaolin Ma, Zhu Chen, Lijuan Chen, Jian Hou, Jian-Qing Mi & Sai-Juan Chen

This PDF file includes:

Supplementary Figures 1 to 11

Supplementary Table 1

Supplementary Figures

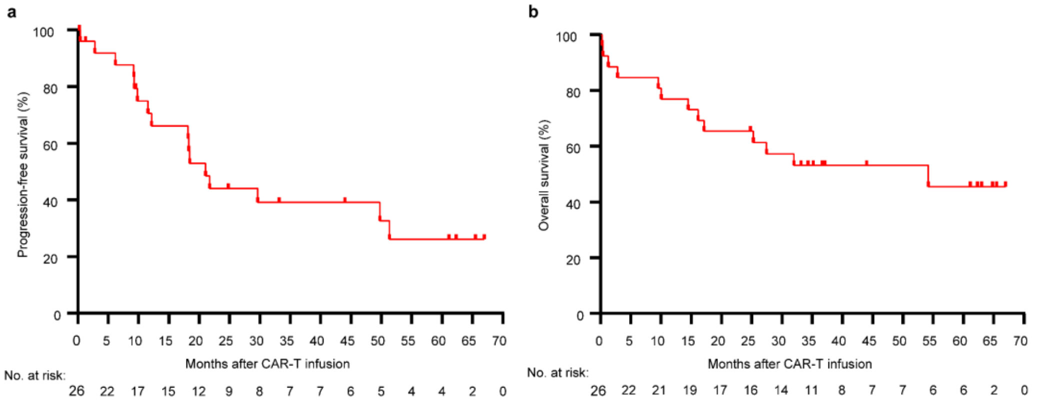

Supplementary Figure 1| Overall clinical response in r/r MM to Cilta-cel.

a, The curve shows the time to progression after infusion of Cilta-cel. **b,** The curve shows overall survival data censored at the time of the last follow-up. Source data are provided as a Source Data file.

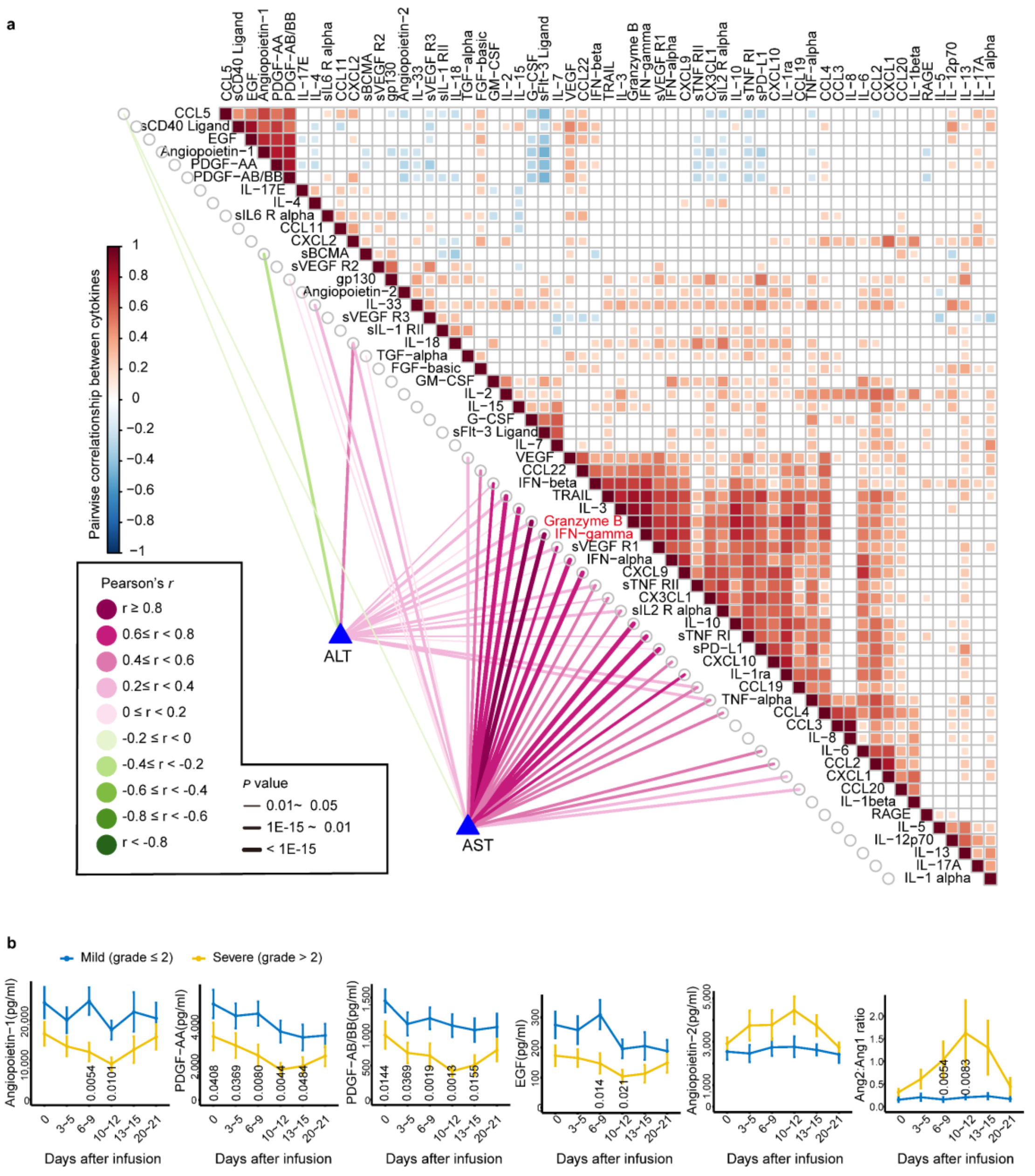

Supplementary Figure 2| Association between clinical indicators and cytokines.

a, This graph shows the correlation between cytokines and hepatic function parameters, aspartate aminotransferase (AST) and alanine aminotransferase (ALT). The heatmap (upper right) exhibits relationship among 61 cytokines and the connection lines (lower left) show relationship between cytokines and AST/ALT levels. Color represents correlation coefficient, and line thickness represents significance. p values are

calculated using two-sided Pearson's *r* correlation. **b**, Based on the NCI-CTCAE v4.03 criteria, patients are divided into severe coagulation group (grade > 2) and mild coagulation group (grade ≤ 2). The curve graphs show the mean values of endothelial-associated molecules in two subgroups, namely patients with mild (blue line, AE grade ≤ 2, n = 10) and severe (yellow line, AE grade ＞ 2, n = 16) toxicity of coagulation, respectively, at indicated time slots after infusion. Data is represented as mean ± SEM. The *p* values are determined using two-sided Mann-Whitney U test, and those with statistically difference ($p < 0.05$) are labeled above the *X* axis. Source data are provided as a Source Data file.

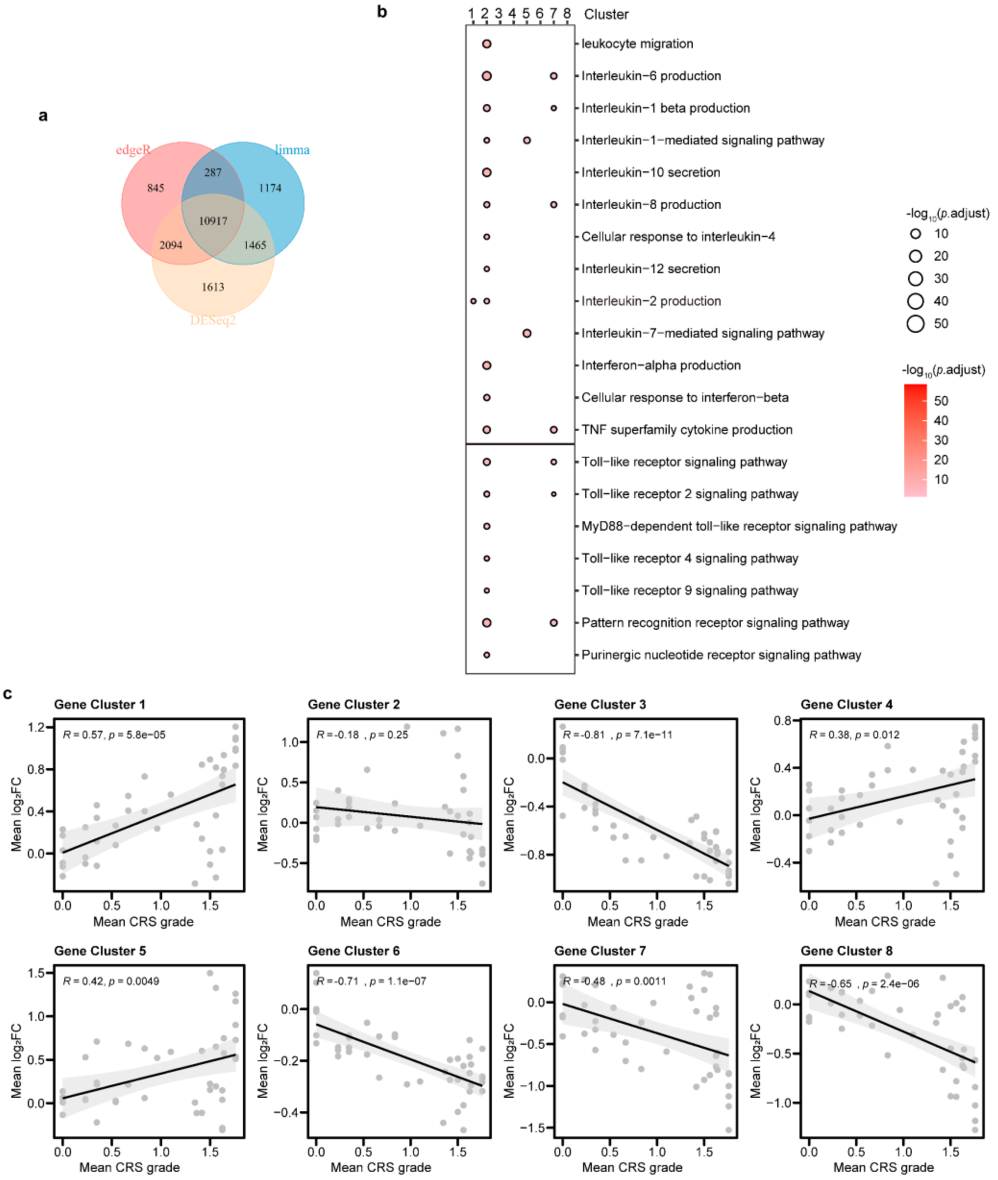

Supplementary Figure 3| Insight of CRS related signaling dysregulation based on the soft clustering method.

a, Upon RNA sequencing, the overlapped differentially expressed genes are selected by three statistical programs: edgeR, Limma, and DESeq2. **b**, Gene ontology analyses of the eight clusters generated by the soft clustering method. The biological processes shown here are the supplements to those in **Figure 4b**. The dot size and darkness represent the significance of pathways in the relevant cluster. An outline of analysis

workflow in **a** and **b** are detailed below. Step 1: Raw expression data filtration to exclude genes with low expression. Step 2: Definition of comparable groups and calculation of DEGs. For each pair of consecutive observation time points, we calculated DEGs independently with three widely recognized R packages: DESeq2, limma, and edgeR. We repeated cyclic computation to calculate DEGs for each pair of observation time points, resulting in the collection of DEGs for each pairwise comparison under each R package, ultimately identifying a total of 10,917 DEGs. Step 3: Soft clustering of DEGs. We used the average expression of the 10,917 DEGs at each time point as input for the Mfuzz package. Step 4: Identification of core genes in each cluster. Genes with an α score > 0.6 were considered core genes and used for subsequent functional enrichment analysis. **c**, Scattered diagrams show the correlation between the mean $\log_2$FC changes of gene transcription and CRS level. *p* values are calculated using two-sided Pearson's *r* correlation. Source data are provided as a Source Data file.

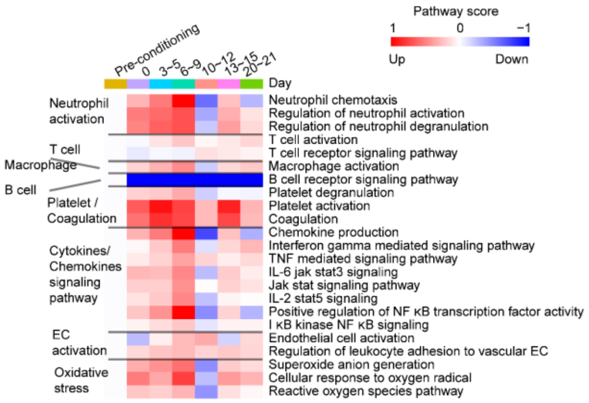

Supplementary Figure 4| Dynamic changes in signaling pathways of significance by gene set score

The heatmap shows dynamic changes of the signaling processes at the indicated time slots upon RNA sequencing. Pathway score is defined as the log_2FC of mean gene set score relative to baseline (blue indicates downregulated; red indicates upregulated). EC indicates endothelial cell. Source data are provided as a Source Data file.

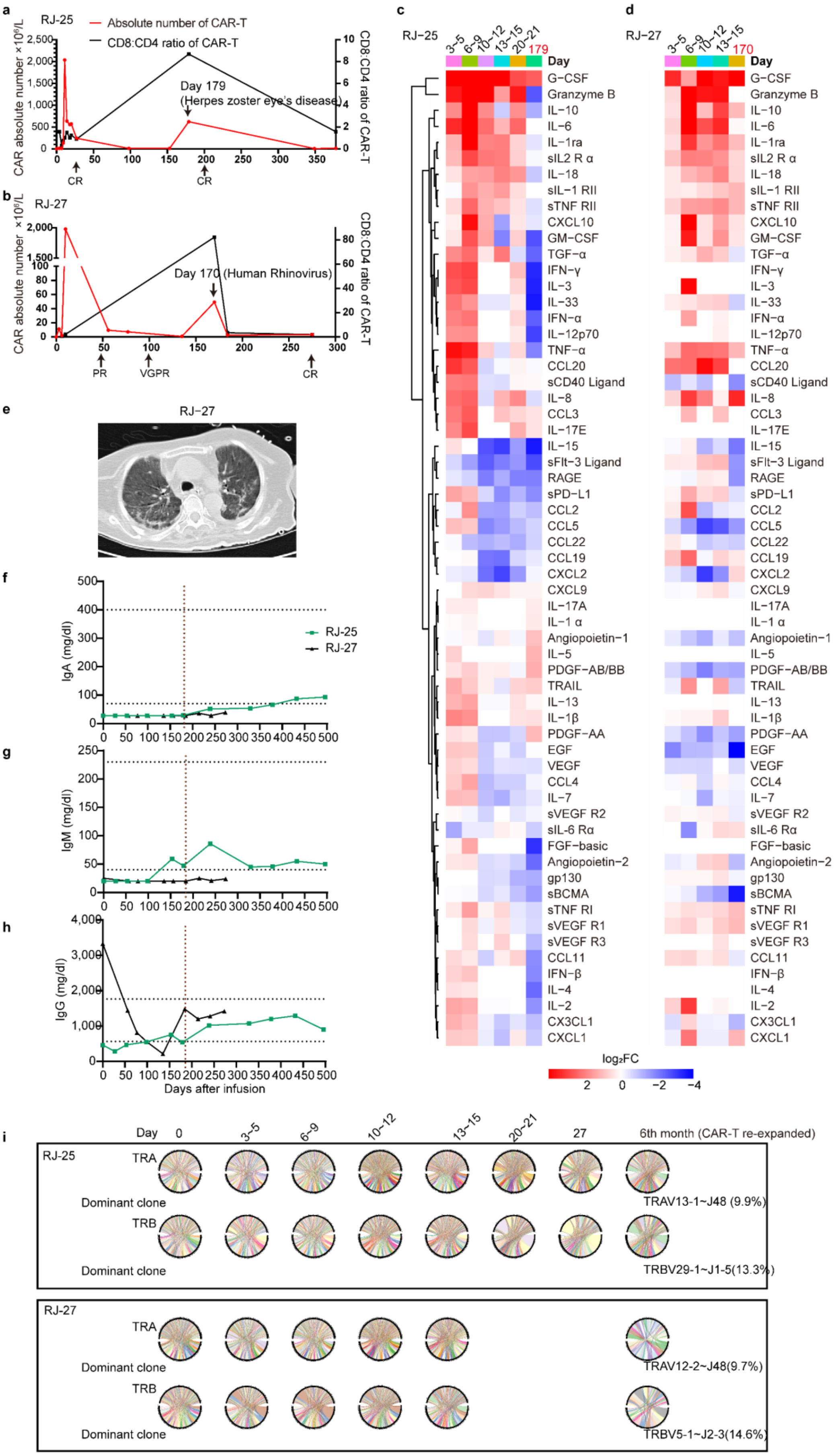
a
RJ-25
Absolute number of CAR-T
CD8:CD4 ratio of CAR-T
CAR absolute number ×10⁶/L
CD8:CD4 ratio of CAR-T
Day 179
(Herpes zoster eye's disease)
CR
CR
b
RJ-27
CAR absolute number ×10⁶/L
CD8:CD4 ratio of CAR-T
Day 170 (Human Rhinovirus)
PR
VGPR
CR
c
RJ-25
3–5
6–9
10–12
13–15
20–21
179
Day
d
RJ-27
3–5
6–9
10–12
13–15
170
Day
G−CSF
Granzyme B
IL−10
IL−6
IL−1ra
sIL2 R α
IL−18
sIL−1 RII
sTNF RII
CXCL10
GM−CSF
TGF−α
IFN−γ
IL−3
IL−33
IFN−α
IL−12p70
TNF−α
CCL20
sCD40 Ligand
IL−8
CCL3
IL−17E
IL−15
sFlt−3 Ligand
RAGE
sPD−L1
CCL2
CCL5
CCL22
CCL19
CXCL2
CXCL9
IL−17A
IL−1 α
Angiopoietin−1
IL−5
PDGF−AB/BB
TRAIL
IL−13
IL−1β
PDGF−AA
EGF
VEGF
CCL4
IL−7
sVEGF R2
sIL−6 Rα
FGF−basic
Angiopoietin−2
gp130
sBCMA
sTNF RI
sVEGF R1
sVEGF R3
CCL11
IFN−β
IL−4
IL−2
CX3CL1
CXCL1
log₂FC
e
RJ-27
f
IgA (mg/dl)
RJ-25
RJ-27
g
IgM (mg/dl)
h
IgG (mg/dl)
Days after infusion
i
Day
0
3–5
6–9
10–12
13–15
20–21
27
6th month (CAR-T re-expanded)
RJ-25
TRA
Dominant clone
TRAV13-1~J48 (9.9%)
TRB
Dominant clone
TRBV29-1~J1-5(13.3%)
RJ-27
TRA
Dominant clone
TRAV12-2~J48(9.7%)
TRB
Dominant clone
TRBV5-1~J2-3(14.6%)

Supplementary Figure 5| Re-expansion of Cilta-cel at the six month in the follow-up of two cases (RJ-25 and RJ-27).

a, b, Kinetic characteristics of Cilta-cel (red line), as well as the CD8:CD4 ratio of Cilta-cel (black line) in the peripheral blood assessed by flow cytometry in the patient RJ-25 (**a**) and RJ-27 (**b**). **c~d**, The heatmaps exhibit dynamic changes of cytokine profile in RJ-25 (**c**) and RJ-27 (**d**) at the given time points, as demonstrated by log_2FC relative to the baseline. Evidently, most of the cytokines didn't remarkably upregulate accompanied by a mild CAR-T expansion at approximately half a year after treatment. **e**, Computed Tomography image of RJ-27's lung**. f~h**, The curve charts illustrate the concentration changes of serum IgA (**f**), IgM (**g**), IgG (**h**) in RJ-25 (green) and RJ-27 (black). **i**, Circle graphs show the polyclonal type of VDJ rearrangement of TCR alpha chain and beta chain in RJ-25 (top panel) and RJ-27 (bottom panel) by means of RNA-sequencing in PBMCs. Source data are provided as a Source Data file.

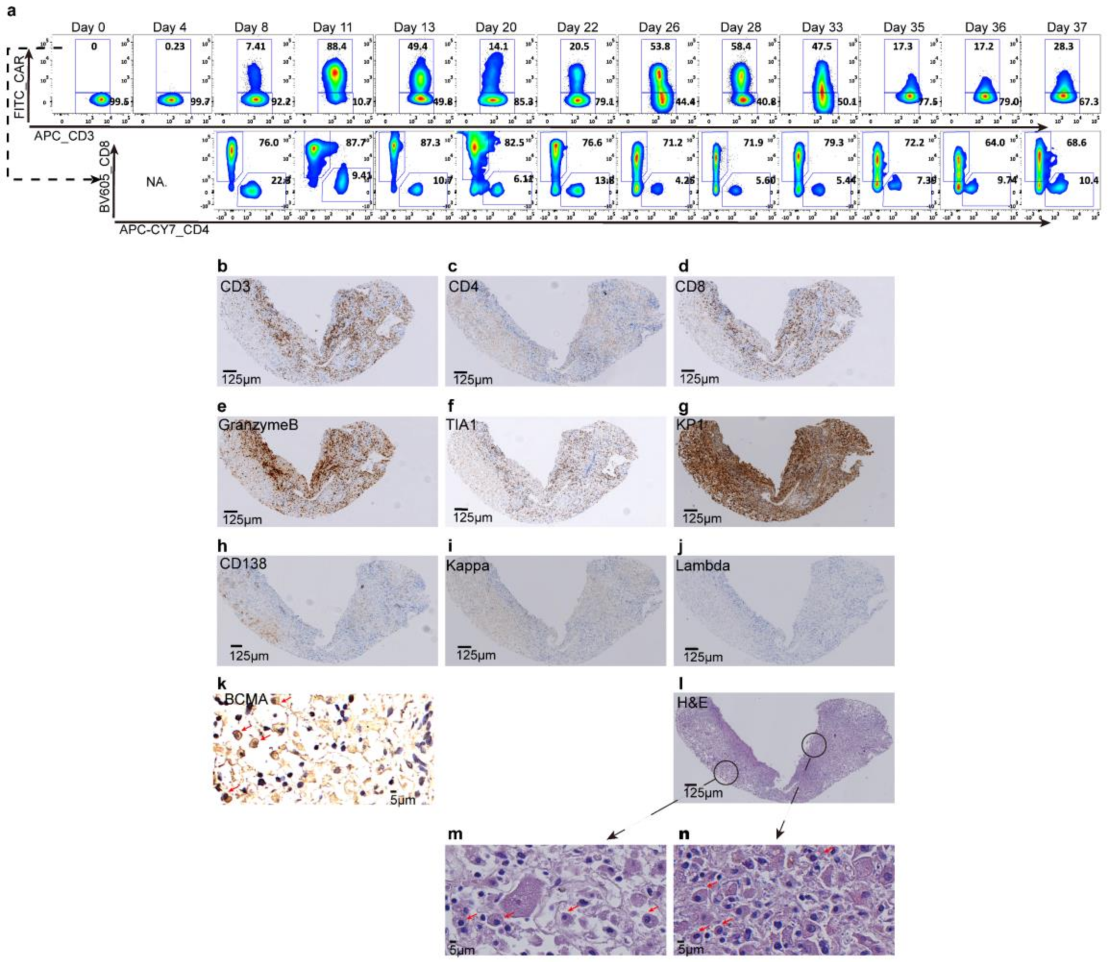

Supplementary Figure 6| Immunophenotyping of the CAR-T and pathological examination of the liver biopsy in RJ-31.

a, Representative flow cytometric plots show the proportions of the entire CAR-T population and its CD4 and CD8 subsets in the peripheral blood after CAR-T infusion**.** **b~k**, Representative immunohistochemistry staining of lymphocyte-related markers CD3 (**b**), CD4 (**c**), CD8 (**d**), toxic particle-related markers Granzyme B (**e**), TIA1 (**f**), macrophage-related marker KP1 (**g**) and plasma cell-related markers CD138 (**h**), kappa (**i**), lambda (**j**), BCMA (**k**) in the liver of RJ-31 during the second CRS. **l~n**, Hematoxylin-eosin staining (H&E) of hepatic tissues. Scattered plasma cells are highlighted in red arrows (**l**, **m**, **n**)**.** NA is abbreviation for “not available”.

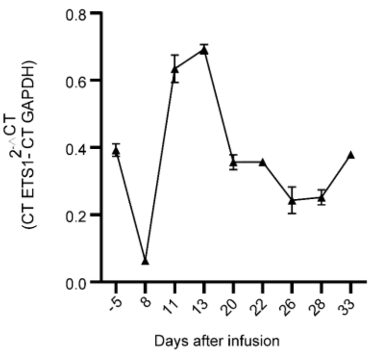

Supplementary Figure 7| *ETS1* transcriptional level of the mononuclear cells in RJ-31

The curved graph shows the mRNA levels of *ETS1* gene, which is detected by RT-qPCR at indicated time points (n = 3 independent experiments). Data is presented as mean ± SEM. Source data are provided as a Source Data file.

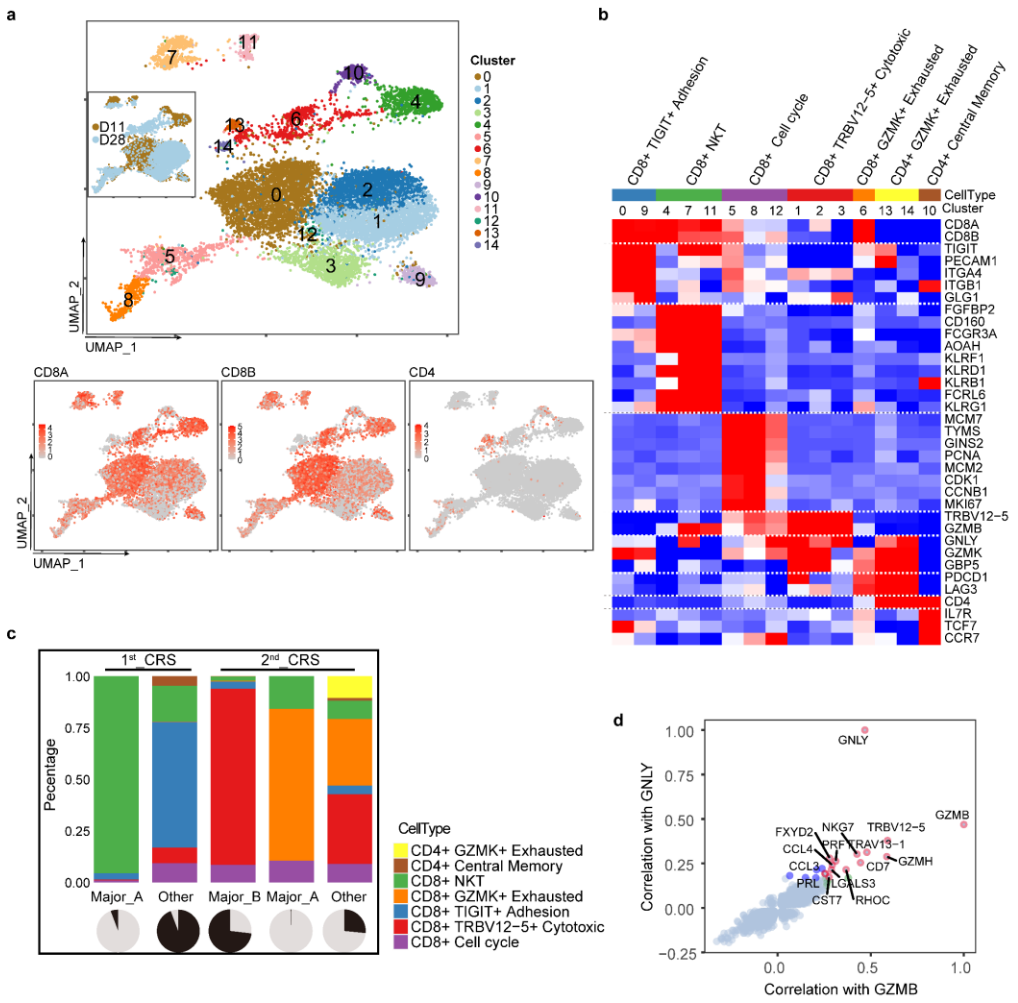

Supplementary Figure 8| 10x Genomics platform based single-cell transcriptome analyses of CAR positive T cell population in RJ-31

a, The UMAP visualization and clustering of 12174 cells captured on day 11 and day 28 (upper). UMAP plots indicate the expression of CD8A, CD8B, CD4 at a single-cell resolution (lower). **b**, Heatmap shows seven CAR-T cell subtypes characterized with specific signature genes with an intent to feature different clusters. **c**, Proportions of cell subtypes in the five purified CAR-T subgroups, in which, clone Major_A represents the major clone in the first CRS and continues to persist in the second CRS, meanwhile clone Major_B referrers the dominance in the second CRS but is undetectable in the first strike. “Other” indicates the other clones in either first CRS or the second time. Pie

charts below depict the percentage that the corresponding clone(s) account for (black). **d**, The top 20 ranked genes significantly associated with *GZMB* and *GNLY* are shown in green and blue dots, respectively. Intersection of those genes are highlighted in red, which are subsequently used to calculate cytotoxic score. Source data are provided as a Source Data file.

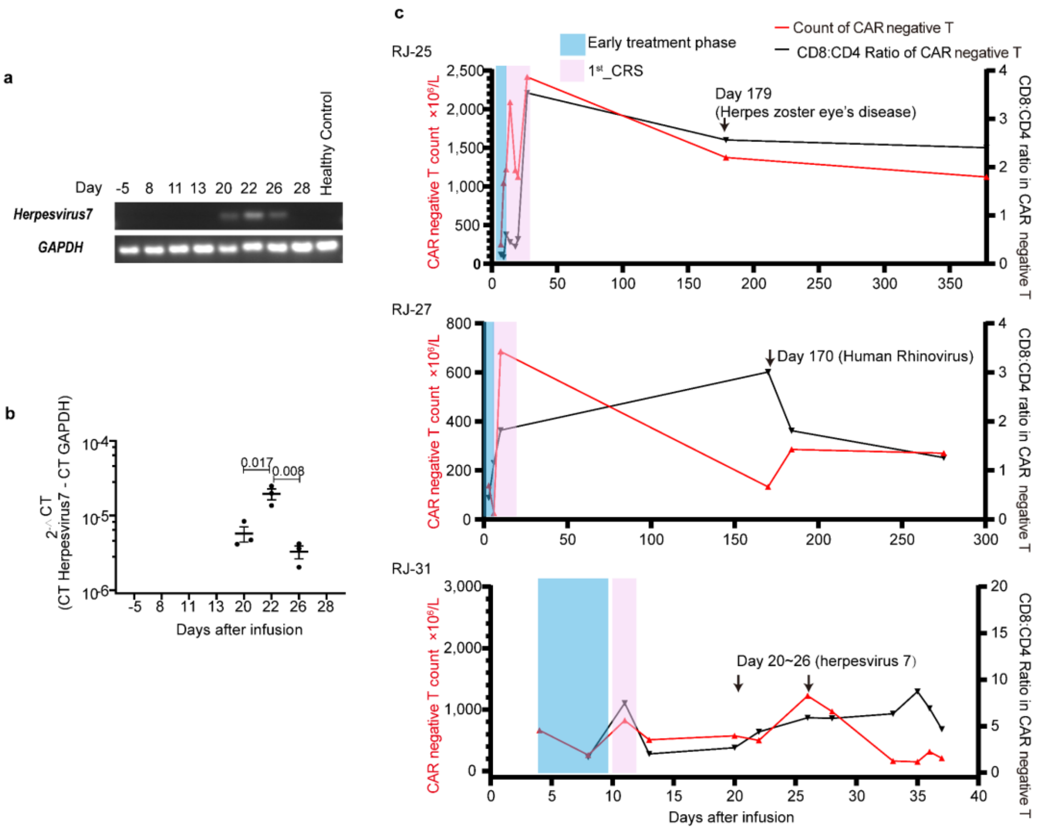

Supplementary Figure 9| Detection of *herpesvirus 7* in RJ-31 and circulating CAR negative T cells kinetics in three patients

a~b, Quantitative measurement of *herpesvirus 7* virus by RT-qPCR in RJ-31. The bands in DNA electrophoresis gel show the fragments specifically for *herpesvirus 7* and a house-keeping gene *GAPDH* following RT-qPCR assay (**a**), and the quantitation of the *herpesvirus 7* transcriptional level relative to *GAPDH* (**b,** n = 3 independent experiments). Data is presented as Mean ± SEM. The *p* values are determined using two-sided *t-test*. **c**, Dynamic changes of the absolute count of CAR negative T cell (red line) and its CD8 to CD4 ratio (black line) in RJ-25, RJ-27 and RJ-31 peripheral blood. The blue legend denotes the post-lymphodepletion phase prior to the CRS outbreak. The red legend refers to the first CRS period after CAR-T infusion. The arrow indicates

the time point of viral infection. Source data are provided as a Source Data file.

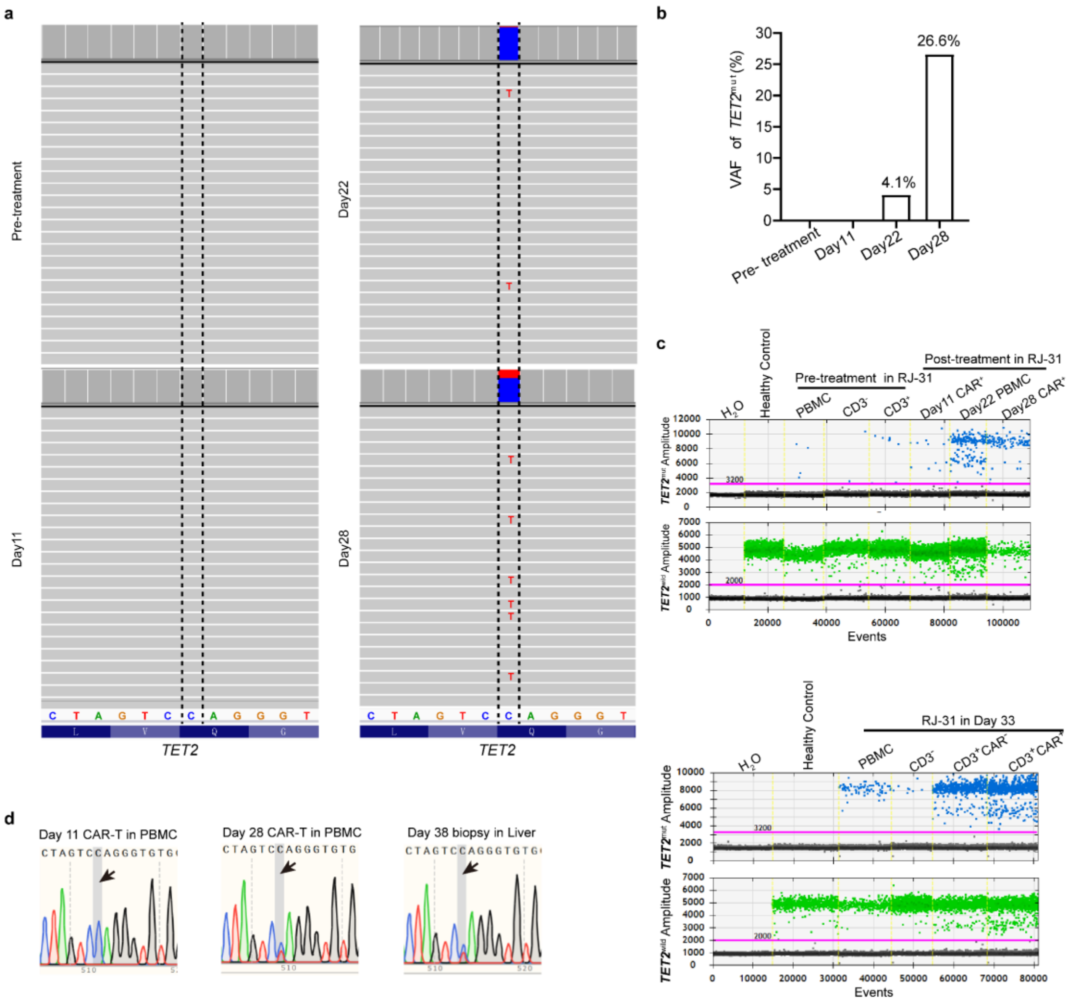

Supplementary Figure 10| Detection of *TET2*^{mut} in RJ-31

a~b, Targeted DNA sequencing identifies a somatic *TET2*^{mut} in the PBMCs of RJ-31 on day 22 and day 28. **a,** Representative view shows the reads overlapping the nonsense mutation (*TET2*^{mut}) at the location highlighted in vertical lines in different samples of RJ-31. The red box indicates mutant, and blue box is reference. **b,** The variant allele fractions (VAFs) of *TET2*^{mut} detected by targeted DNA sequencing are shown by the columns. **c,** Representative ddPCR droplet plot shows the mutant (*TET2*^{mut}) and wild-type (*TET2*^{wild}) populations for each sample. **d**, Detection of *TET2*^{mut} in the circulating CAR-T or the biopsy in liver of RJ-31 by Sanger sequencing. The nonsense point

variation framed in grey formed a stop codon. Source data are provided as a Source Data file.

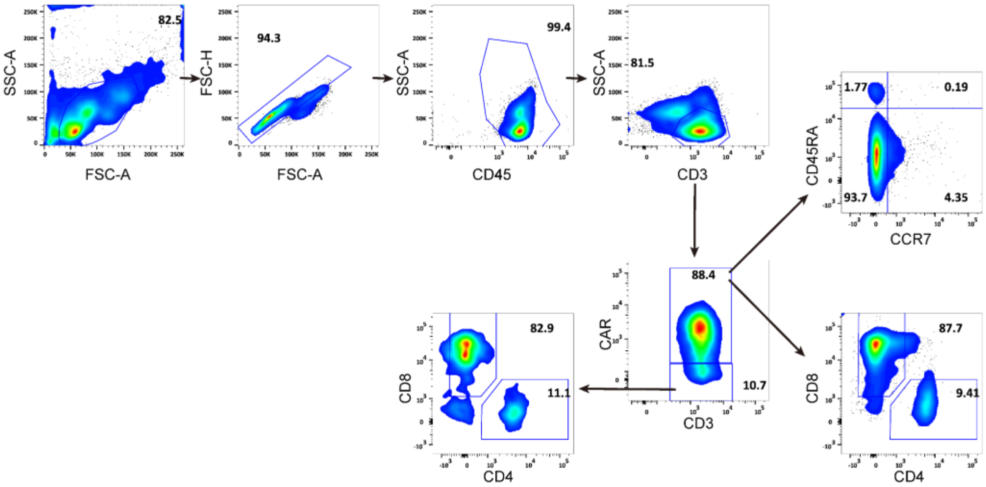

Supplementary Figure 11| Flow cytometry gating strategy for immunophenotyping.

Representative flow cytometry plots showing the gating strategy used to analysis the immunophenotype of T cells.

Supplementary Table 1. Summary of primers sequences involved in Lentiviral vector Integration site experiment

Name	Sequence (5'-3')
Primer 1	AATAAAGCGGTCCTTGAGTGCT
UMI Sense	GACCCGGGAGATCTGAATTCAGTGGCACAGCAGTTAGG
UMI Antisense	CCTAACTGCTGTGCCACTGAATTCAGATC
Primer 2	AGTAGTGTGTGGTCGTCTGT
Primer 3	GACCCTGGTGATCTGAATTC
Primer 4	CAAGCAGAAGACGGCATACGAGATCGGTCTCGGCATTCCTGCTGAACCGCTCTTCCGATCTAGTGGCACAGCAGTTAGG
