# Supplementary material for: Neutrophil activation and clonal CAR-T re-expansion underpinning cytokine release syndrome during ciltacabtagene autoleucel therapy in multiple myeloma
Source: Nat Commun. 2024 Jan 8;15:360. doi: 10.1038/s41467-023-44648-3 (PMC10774397; doi:10.1038/s41467-023-44648-3)
Supplement: Supplementary file 3 — Description of Additional Supplementary Files [file 41467_2023_44648_MOESM3_ESM.pdf]

Description of Additional Supplementary Files

File Name: Supplementary Data 1
Description: Summary of the assays on the single patient basis

File Name: Supplementary Data 2
Description: Adverse effects of Cilta-cel and their managements

File Name: Supplementary Data 3
Description: Correlations between cytokines and CRS level
