# Supplementary material for: Neutrophil activation and clonal CAR-T re-expansion underpinning cytokine release syndrome during ciltacabtagene autoleucel therapy in multiple myeloma
Source: Nat Commun. 2024 Jan 8;15:360. doi: 10.1038/s41467-023-44648-3 (PMC10774397; doi:10.1038/s41467-023-44648-3)
Supplement: Supplementary file 7 — Reporting Summary [file 41467_2023_44648_MOESM7_ESM.pdf]

nature portfolio

Corresponding author(s): Jian-Qing Mi; Sai-Juan Chen

Last updated by author(s): 2023/12/1

Reporting Summary

Nature Portfolio wishes to improve the reproducibility of the work that we publish. This form provides structure for consistency and transparency in reporting. For further information on Nature Portfolio policies, see our Editorial Policies and the Editorial Policy Checklist.

Statistics

For all statistical analyses, confirm that the following items are present in the figure legend, table legend, main text, or Methods section.

n/a	Confirmed	
☐	☒	The exact sample size (*n*) for each experimental group/condition, given as a discrete number and unit of measurement
☐	☒	A statement on whether measurements were taken from distinct samples or whether the same sample was measured repeatedly
☐	☒	The statistical test(s) used AND whether they are one- or two-sided<br>*Only common tests should be described solely by name; describe more complex techniques in the Methods section.*
☒	☐	A description of all covariates tested
☒	☐	A description of any assumptions or corrections, such as tests of normality and adjustment for multiple comparisons
☐	☒	A full description of the statistical parameters including central tendency (e.g. means) or other basic estimates (e.g. regression coefficient) AND variation (e.g. standard deviation) or associated estimates of uncertainty (e.g. confidence intervals)
☐	☒	For null hypothesis testing, the test statistic (e.g. *F*, *t*, *r*) with confidence intervals, effect sizes, degrees of freedom and *P* value noted<br>*Give P values as exact values whenever suitable.*
☒	☐	For Bayesian analysis, information on the choice of priors and Markov chain Monte Carlo settings
☒	☐	For hierarchical and complex designs, identification of the appropriate level for tests and full reporting of outcomes
☐	☒	Estimates of effect sizes (e.g. Cohen's *d*, Pearson's *r*), indicating how they were calculated

Our web collection on statistics for biologists contains articles on many of the points above.

Software and code

Policy information about availability of computer code

Data collection	1.Sequencing: Illumina NovaSeq 6000;<br>2.Flow cytometry: LSRII flow cytometer (Becton Dickinson, Franklin Lakes, NJ, USA);<br>3. Luminex: Luminex X-200 instrument;<br>4. qPCR: ViiATM 7 system (Life technologies);<br>5. ddPCR: Bio-Rad QX200 system;
Data analysis	FlowJo software V10 (TreeStar, Ashland, OR, USA);Milliplex analyst software;R version 4.0.3; GraphPad Prism version 8.0.2; QuantaSoft Version 1.7.4.

For manuscripts utilizing custom algorithms or software that are central to the research but not yet described in published literature, software must be made available to editors and reviewers. We strongly encourage code deposition in a community repository (e.g. GitHub). See the Nature Portfolio guidelines for submitting code & software for further information.

Data

Policy information about availability of data

All manuscripts must include a data availability statement. This statement should provide the following information, where applicable:

- Accession codes, unique identifiers, or web links for publicly available datasets
- A description of any restrictions on data availability
- For clinical datasets or third party data, please ensure that the statement adheres to our policy

All data generated, analysed and availability during this study are included in this manuscript (and its supplementary information files). Raw sequencing data generated during this study have been deposited in the Genome Sequence Archive in National Genomics Data Center, China National Center for Bioinformation / Beijing Institute of Genomics, Chinese Academy of Science (https://ngdc.cncb.ac.cn/gsa-human), with accession number 'GSA-Human: HRA005381' [https://ngdc.cncb.ac.cn/gsa-human/browse/HRA005381]. Access can be obtained by approval via the Data Access Committee of the GSA-human database (https://ngdc.cncb.ac.cn/gsa-human/document/GSA-Human_Request_Guide_for_Users_us.pdf). Original data for graphs is provided in the Source Data file. Source data are provided with this paper.

Research involving human participants, their data, or biological material

Policy information about studies with human participants or human data. See also policy information about sex, gender (identity/presentation), and sexual orientation and race, ethnicity and racism.

Reporting on sex and gender	Biological sex for all participants is provided in Table 1.
Reporting on race, ethnicity, or other socially relevant groupings	Race, ethnicity, or other socially relevant groupings are not addressed in this study.
Population characteristics	All participant characteristics are provided in Table 1.
Recruitment	This study included all subjects with available specimens in our clinical center, Rui Jin Hospital affiliated with Shanghai Jiao Tong University School of Medicine (referred to as RJ hereafter). The participating sites of the phase I trial of ciltacabtagene autoleucel comprised four centers with one in the West China and the other three in the East. The East area with RJ as the leading site administrated Cilta-cel treatment in 17 patients, of whom, 16 patients' sequential serum samples were obtained while in one case the sample was unavailable. The phase II trial was conducted in eight centers. Each site was in charge of its own patient samples collection. As of manuscript preparation, RJ had treated 10 phase II patients whose serum samples were entirely investigated. Therefore, 26 patients composed of 16 from phase I and 10 from phase II were investigated in this study without any selection bias.
Ethics oversight	Participants in this study were from phase I (NCT03090659, ChiCTR-ONH-17012285) and phase II (NCT03758417) clinical trials of ciltacabtagene autoleucel in China with Rui Jin Hospital affiliated with Shanghai Jiao Tong University School of Medicine as the leading site. All participants provided written informed consent.

Note that full information on the approval of the study protocol must also be provided in the manuscript.

Field-specific reporting

Please select the one below that is the best fit for your research. If you are not sure, read the appropriate sections before making your selection.

☒ Life sciences ☐ Behavioural & social sciences ☐ Ecological, evolutionary & environmental sciences

For a reference copy of the document with all sections, see nature.com/documents/nr-reporting-summary-flat.pdf

Life sciences study design

All studies must disclose on these points even when the disclosure is negative.

Sample size	This study included 26 subjects with available specimens in our clinical center.
Data exclusions	No data were excluded from any analysis in this study.
Replication	The study declared two key findings. One finding regarding CRS development mechanism was generated by Luminex-based serum cytokine detection and RNA sequencing. The two techniques were respectively carried out in 26 and 9 patients. Thus, this part of results were reproducible. The other finding is relevant to CAR-T re-expansion that was observable in three patients. Particularly, one patient carried a somatic TET2 mutation which ultimately led to a clonal expansion of CAR-T cells under the circumstance of virus infection. Such a rare case may not be reproducible.
Randomization	The participating sites of the Legend-2 trial comprised four centers with one in the West China and the other three in the East (NCT03090659, ChiCTR-ONH-17012285). The East area with our center as the leading site conducted Cilta-cel treatment in 17 patients, of whom, 16 patients' sequential serum samples were obtained while in one case the sample was unavailable. The CARTIFAN-1 trial was conducted in eight centers

(NCT03758417). Each was in charge of its own patient samples collection. As of manuscript preparation, our center had treated 10 phase II patients whose serum samples were entirely investigated. Therefore, 26 r/r MM patients with available specimens in our clinical center were included in this study without any selection bias.

Blinding

We divided patients into mild and severe CRS groups according to the CRS grading consensus recommendation. Mild CRS was defined as grade 2 or lower, and severe CRS was higher than grade 2. Hence, blinding was not relevant to this study.

Reporting for specific materials, systems and methods

We require information from authors about some types of materials, experimental systems and methods used in many studies. Here, indicate whether each material, system or method listed is relevant to your study. If you are not sure if a list item applies to your research, read the appropriate section before selecting a response.

Materials & experimental systems

n/a	Involved in the study	
☐	☒	Antibodies
☒	☐	Eukaryotic cell lines
☒	☐	Palaeontology and archaeology
☒	☐	Animals and other organisms
☐	☒	Clinical data
☒	☐	Dual use research of concern
☒	☐	Plants

Methods

n/a	Involved in the study	
☒	☐	ChIP-seq
☐	☒	Flow cytometry
☒	☐	MRI-based neuroimaging

Antibodies

Antibodies used

PE Mouse Anti-Human CD45 (BD, Cat: 555483; Clone: HI30; Lot: 9337233)、 APC Mouse Anti-Human CD3 (BD, Cat:555335; Clone: UCHT1; Lot: 7200698)、APC-Cy™7 Mouse Anti-Human CD4 (BD, Cat:557871; Clone: RPA-T4; Lot: 7348612)、Hu CD8 BV605 (BD, Cat:564116; Clone: SK1; Lot: 8137958)、BV711 Mouse Anti-Human CD45RA (BD, Cat:563733; Clone: HI100; Lot: 8073653)、BV421 Mouse Anti-Human CD197 (CCR7) (BD, Cat:562555; Clone: 150503; Lot: 7355865)、FITC-Labeled Human BCMA / TNFRSF17 Protein (ACRO Biosystems, Cat: BCA-HF254; Lot: FL894-20BHF1-VK)

Validation

The antibodies used in this study are commercially available and were used for the applications validated by manufacturers. Validation statements can be found on manufacturer websites.

Clinical data

Policy information about clinical studies

All manuscripts should comply with the ICMJE guidelines for publication of clinical research and a completed CONSORT checklist must be included with all submissions.

Clinical trial registration

NCT03090659, ChiCTR-ONH-17012285 and NCT03758417

Study protocol

All the evaluated patients underwent cyclophosphamide-based (with or without fludarabine) lymphodepletion therapy followed by Cilta-cel infusion at a median dose of 0.595x106 CAR-T cells/kg. The details of the study protocols are available in the following websites (https://clinicaltrials.gov/study/NCT03090659; https://www.chictr.org.cn/showproj.html?proj=20671; https://clinicaltrials.gov/study/NCT03758417)

Data collection

Study participant recruitment and sample collection took place in Ruijin Hospital Affiliated to Shanghai Jiao Tong University School of Medicine between April 2017 and November 2020. Data collection and analysis were finalized in December 2022.

Outcomes

As of the manuscript preparation, the overall response rate of the 26 cases was 88.5%, with 80.8% obtaining complete responses . The progression-free survival rates were 39.2% at 3 year and 26.1% at 5 year; the overall survival rates were 53.1% at 3 year and 45.5% at 5 year. CRS was observed in all patients. 34.6% patients had Grade 1~2 CRS and 65.4% suffered Grade 3 or worse.

Flow Cytometry

Plots

Confirm that:

☒ The axis labels state the marker and fluorochrome used (e.g. CD4-FITC).

☒ The axis scales are clearly visible. Include numbers along axes only for bottom left plot of group (a 'group' is an analysis of identical markers).

☒ All plots are contour plots with outliers or pseudocolor plots.

☒ A numerical value for number of cells or percentage (with statistics) is provided.

Methodology

Sample preparation	PBMC were collected and isolated from peripheral blood,CAR-T immunophenotyping was detected using fluorochrome-conjugated antibodies. After incubating with the antibodies as indicated by the manufacturer for 30 minutes at +4°C, PBMCs were washed in PBS and acquired to the flow cytometer.
Instrument	LSRII flow cytometer (Becton Dickinson, Franklin Lakes, NJ, USA)
Software	FlowJo software V10 (TreeStar, Ashland, OR, USA)
Cell population abundance	CAR positive cells were selected by flow-based sorting to achieve nearly 95% purity. And the purity was checked for CAR expression by flow cytometry.
Gating strategy	Gated upon singlets (FSC-A/SSC-A and FSC-H/FSC-A gates), T cells (CD45+ and CD3+) were selected. And then CAR positive and CAR negative T cells were selected from T cells population. Meanwhile, phenotypic markers (CD8/CD4 or CCR7/CD45RA) were used for in-depth T-cell immunophenotyping.

☒ Tick this box to confirm that a figure exemplifying the gating strategy is provided in the Supplementary Information.
